# The prognostic role of sex and hemoglobin levels in patients with oral tongue squamous cell carcinoma

**DOI:** 10.3389/fonc.2022.1018886

**Published:** 2022-11-15

**Authors:** Marta Tagliabue, Oriana D’Ecclesiis, Rita De Berardinis, Aurora Gaeta, Chiara Martinoli, Andrea Fausto Piana, Fausto Maffini, Sara Gandini, Mohssen Ansarin, Susanna Chiocca

**Affiliations:** ^1^ Department of Otolaryngology Head and Neck Surgery, European Institute of Oncology (IEO) Istituto di Ricovero e Cura a Carattere Scientifico (IRCCS), Milan, Italy; ^2^ Department of Biomedical Sciences, University of Sassari, Sassari, Italy; ^3^ Department of Experimental Oncology, European Institute of Oncology (IEO) Istituto di Ricovero e Cura a Carattere Scientifico (IRCCS), Milan, Italy; ^4^ Department of Medicine, Surgery and Pharmacy, University of Sassari, Sassari, Italy; ^5^ Division of Pathology, European Institute of Oncology Istituto di Ricovero e Cura a Carattere Scientifico (IRCCS), Milan, Italy

**Keywords:** sex, gender, head and neck cancer, head and neck squamous cell carcinoma, tongue cancer, hemoglobin, neutrophil lymphocyte ratio, survival

## Abstract

**Background:**

Women and men differ genetically, biologically (sex) and by social construct (gender), possibly impacting on prognostic factors in predicting cancer survival. Hemoglobin levels and immune system activation are players acting in this scenario which could play a role in partly determining prognosis between patients of different sex/gender (S/G). Here, we investigate these factors in patients affected by tongue squamous cell carcinoma.

**Methods:**

This is an observational retrospective cohort study. We collected tongue cancer patients’ clinical data, including hemoglobin levels and neutrophil lymphocyte ratio (NLR). Overall survival (OS) and disease-free survival (DFS) were compared between women and men considering confounding and prognostic factors in multivariate Cox proportional hazard models. Stratified analyses were also conducted by sex and tumor stage.

**Result:**

576 patients, 39.9% women and 60.1% men, were found eligible for the analysis. Men were more often smokers (p<0.001), alcohol consumers (p<0.001), overweight or obese (p<0.001) and undergoing radiotherapy (p=0.002). In multivariate models for stage I-II, men showed half risk of death and relapse compared to women (HR=0.44; 95%CI 0.24-0.81, p=0.009; HR=0.55; 95%CI 0.34-0.87, p=0.01, for OS and DFS respectively). Moreover, low hemoglobin levels appeared to be an independent prognostic factor for women but not for men in terms of both OS and DFS. Specifically, women with low hemoglobin levels showed a worse tumor outcome (HR=2.66; 95%CI 1.50-4.70; HR=2.09; 95%CI 1.24-3.53, for OS and DFS respectively). Low hemoglobin levels appeared to be a poor OS prognostic factor for women at stage I-II (p<0.004) but not for men (p=0.10). Women with advanced stage tumors, NLR>2.37, who did not performed Radiotherapy and with depth of invasion (DOI)> 10 were associated with a significant increase in relapse and death (all p<0.05).

**Conclusion:**

In our cohort of patients with oral tongue squamous cell carcinoma, men present better OS and DFS than women with early stages tumors. Low hemoglobin level was an independent prognostic factor for women, especially at early-stage tumors. For advanced stages (III-IV), sex is not a significant factor related to patients’ prognosis.

## Introduction

Recently, more literature is emphasizing the diversity in prognosis and response to treatment between men and women diagnosed with similar stage tumors. Genetic differences between men and women lead to a difference in cancer prevalence, manifestation, and response to treatment (1). These differences are reported for women and men also across different disorders (1). For example, women are more likely to develop Alzheimer’s disease, autoimmune disorders, or depression (2), while men of all races develop cancer with an overall worse survival outcome at all ages and worldwide (2–4). These differences may be due to an increased exposure in men to classical risk (as tobacco and alcohol) (5) and prognostic factors but also because of sex-specific biology (XX vs XY chromosomes) (3, 6). As known, sex chromosomes can regulate cellular pathways in sex specific ways, through different proteins and receptors, cell growth regulation, inflammatory response, and immune defense activation (2, 3, 7).

Despite these relevant elements, studies on sex as a biological variable are still lacking especially in head and neck cancers (HNC) (8, 9).

Higher mortality rates for men compared to women are reported in some HNC studies (10, 11). However, these differences seem to be less evident when patients are treated in third level centers for HNC underscoring the importance and value of high-level daily cancer practice for cancer remission (12, 13).

HNC includes different anatomical sites: the oral cavity, the pharynx, the larynx, the nasal cavity, the paranasal sinuses, the salivary glands, and the thyroid. These entities differ significantly in risk and prognostic factors, etiology, histology, therapeutic management, and oncological outcomes (5).

Ninety per cent of HNCs are squamous cell carcinoma (HNSCC) and more than 70% of HNSCC affect men (14, 15). Moreover, men represent more than 80% of the laryngeal subgroup but the difference in incidence by sex fades in the oral cavity (16).

In 2018 for oral cavity cancers, the worldwide age-standardized rates (ASRs) of new cases were 5.8 for men and 2.3 for women per 100 000 persons, the mortality reported was 2.8 for men and 1.2 for women (17).

According to Global Cancer Observatory data, the incidence of oral cavity cancers, including tongue cancers, is set to grow in all countries from 2020 to 2040 (18). This increase is described for both sexes although apparently in women the growth seems to be greater than in the men (18).

Nowadays, the risk of developing oral cancer from precancerous mucosal lesions appears to affect women more than men and is well documented in the literature, but there are only a few studies evaluating the role of sex in oral tongue cancer (19, 20).

Moreover, in HNC, low hemoglobin (Hb) levels are associated with poor prognosis and greater incidence causing chronic inflammation (21) and decreasing response to treatment (22, 23).

The prevalence of low hemoglobin levels in women is significantly higher than in men for physiological pathways and for their everyday life choices, as women are more inclined to have vegetarian dietary habits that could lead to lower hemoglobin levels (Hb < 13,5 g/dL for men, and Hb < 12 g/dL for women (24–27). Furthermore, anemia is associated with socio-economic factors, revealing the cultural aspects influencing this biomarker (28–31). Often circulating biomarkers reflect innate, evolved and hormonal factors together with social, experiential and cultural habits (32, 33). Therefore, we will use the term sex/gender (S/G) in the manuscript when referring to the complex relationship between them. In fact, while the term “sex” refers to genetic/biological differences, gender considers the sexual identity of the individual and everyday life choices, as reported by the World Health Organization (WHO): “Gender identity refers to a person’s deeply felt, internal and individual experience of gender, which may or may not correspond to the person’s physiology or designated sex at birth” (34).

Finally, leukocytes are functionally diverse in HNC patients compared to healthy subjects (35). Inflammatory responses are connected both to tumor suppression and progression, thus high neutrophil lymphocyte ratio (NLR) is associated with poor prognosis in oral cancer and several other cancer sites (36–40).

The aim of our work is to investigate possible differences in tongue cancer in terms of survival and prognostics factors, based on patients’ sex/gender (S/G). Together with other well-known prognostic factors, we considered hemoglobin and NLR comparing men and women, as they are measurements routinely collected from patients prior to any treatment and reported to be related to oral cancer prognosis (22, 36).

## Material and methods

Five hundred and seventy-six patients, 230 women and 346 men, with primary diagnosis of oral tongue squamous cell carcinoma (OTSCC) and primary surgical treatment received at the European Institute of Oncology, IRCCS (IEO) between 2000 and 2018 were included in the study cohort.

All the data analyzed were retrospectively extracted from electronic medical records.

Smoking and alcohol status at diagnosis were collected to classify patients as current, former, and never-smoking/drinking.

The diagnosis of low hemoglobin levels was based on the WHO standardized cut-off values: Hb < 13,5 g/dL for men, and Hb < 12 g/dL for women (24, 25).

Also, the information on family cancers histories (head and neck and other cancers) and NLR were collected.

All tumor stages according the new VIII TNM edition (41) were included. All cases without data on tumor depth of invasion (DOI) were reviewed by a head and neck pathologist (FM) according to the current definition of DOI (42). Patients with involvement of extrinsic tongue musculature by disease were considered with a DOI greater than at least 10 mm  (43). All patients underwent surgery glossectomies according to Ansarin et al. classification (44) and radiotherapy (RT) or radio-chemotherapy (RCT) according to the pathological tumor stage and following NCCN international guidelines (45).

Follow-up for all patients were updated to assess patients’ medical status at the last clinical evaluation and contact. Patient deaths and their possible causes were assessed using the Italian national death registers.

Ethics Committee approval: IEO 225.

### Statistical methods

We calculated the median and interquartile range (IQR) for continuous variables and absolute and relative frequencies as summary measures of categorical variables. Based on the nature of variables, Fishers-Exact tests, Wilcoxon Rank tests or the Kruskal-Wallis rank sum test were performed to investigate association of S/G with clinical characteristics and biomarkers. Disease-free survival (DFS) was calculated from date of surgery to disease progression or death (event), or last follow-up (censored). Overall survival (OS) was calculated from date of surgery to death (event) or the last follow-up (censored). DFS and OS curves were estimated with the Kaplan-Meier method, and survival distributions were compared using Log-Rank test.

Multivariate Cox proportional hazards models have been used to study both the prognostic role of S/G and possible other independent prognostic factors, including serum biomarkers. We conducted stratified analyses by S/G and tumor stage.

Results are presented as hazard ratios (HRs) with 95% confidence intervals (95%CIs), adjusting for confounder and important prognostic factors. For all analyses, two-tailed P<0.05 was considered statistically significant. The statistical analyses were performed with R software, version 4.1.1.

## Results

Clinical-pathological and tumor characteristics of the whole study population were reported on **Table 1**.

**Table 1:** Patients and tumor characteristics (N=576 patients, 39.9% women and 60.1% men).

		Overall (N=576)	Women (N=230)	Men (N=346)	P value*
Age, median (IQR)		56 (44-68)	56 (44-71)	56 (45-66)	0.16
BMI, median (IQR)		25 (22-28)	24 (20.9-27)	25 (23-28)	**<0.001**
Alcohol status, n (%)	No	288 (50.0)	159 (69.1)	129 (37.3)	**<0.001**
	Yes	265 (46.0)	63 (27.4)	202 (58.4)	
	Ex	14 (2.4)	4 (1.75)	10 (2.9)	
	Missing	9 (1.6)	4 (1.75)	5 (1.4)	
Smoking status, n (%)	No	208 (36.1)	130 (56.5)	78 (22.5)	**<0.001**
	Yes	199 (34.5)	48 (20.9)	151 (43.6)	
	Ex	162 (28.1)	49 (21.3)	113 (32.6)	
	Missing	7 (1.3)	3 (1.3)	4 (1.3)	
History of family tumor n (%)	No	546 (94.8)	225 (97.8)	321 (92.8)	0.99
	Yes	11 (1.9)	4 (1.7)	7 (2.0)	
	Missing	19 (3.3)	1 (0.5)	18 (5.2)	
Low hemoglobin levels, n (%)	No	491 (85.2)	197 (85.6)	294 (85.0)	0.72
	Yes	67(11.6)	29 (12.6)	38 (11.0)	
	Missing	18 (3.2)	4 (1.8)	14 (4.0)	
	Free	489 (84.9)	194 (84.3)	295 (85.3)	0.51
Margins	Positive	14 (2.4)	8 (3.5)	6 (1.7)	
	Close (< 1 mm)	72 (12.5)	28 (12.2)	44 (12.7)	
	Missing	1 (0.2)	0 (0)	1 (0.3)	
	≤5 mm	142 (24.6)	74 (32.2)	68 (19.7)	**0.002**
DOI (mm)	>5 and ≤ 10 mm	145 (25.2)	61 (26.5)	84 (24.3)	
	>10 and ≤ 20 mm	285 (49.5)	94 (40.9)	191 (55.2)	
	>20 mm	4 (0.7)	1 (0.4)	3 (0.8)	
	No	346 (60.1)	156 (67.8)	190 (54.9)	**0.002**
RT, n (%)	Yes	230 (39.9)	74 (32.2)	156 (45.1)	
	No	477 (82.8)	195 (84.8)	282 (81.5)	0.36
RT+CT, n (%)	Yes	99 (17.2)	35 (15.2)	64 (18.5)	
Vascular invasion, n (%)	No	547 (95.0)	222 (96.5)	325 (93.9)	0.20
	Yes	29 (5.0)	8 (3.5)	21 (6.1)	
Perineural infiltration, n (%)	No	490 (85.1)	196 (85.2)	294 (85.0)	0.99
	Yes	86 (14.9)	34 (14.8)	52 (15.0)	
	I	130 (22.6)	67 (29.1)	63 (18.2)	**0.01**
Stage, n (%) VIII edition	II	94 (16.3)	40 (17.4)	54 (15.6)	
	III	176 (30.5)	56 (24.3)	120 (34.7)	
	IVa	102 (17.7)	39 (16.9)	63 (18.2)	
	IVb	74 (12.9)	28 (12.3)	46 (13.3)	
Preoperatory hemoglobin, median (IQR)		14.3 (13.2-15.2)	13.6 (12.7-14.3)	14.8 (13.8-15.5)	**<0.001**
NLR, median (IQR)		2.37 (1.81-3.23)	2.31 (1.88-3.02)	2.39 (1.77-3.41)	0.52

*P value for testing differences between women and men in terms of characteristics in table.

BMI, body mass index; RT, radiotherapy; RT+CT, radiotherapy+chemotherapy; NLR, neutrophil lymphocyte ratio; IQR, interquartile range.

Of 576 patients, 230 (39.9%) were women and 346 (60.1%) men, and the median follow-up was 7.63 years (y) (4.07y-12.2y). The median survival was 12.3y (10.2y-16y) and the DFS median was 4.85 y (3.31-7.06y).

Out of 576 patients, 248 (43%) had a recurrence of loco/regional and/or distant disease, of these 37% recovered after a second treatment (**Supplementary Table S1**).

Of 576 patients 246 (43%) died and of these, 65% died for tongue cancer (27% of the whole cohort) (**Supplementary Table S2**).

We observed statistically significant differences in patients upon considering sex and lifestyle habits: men patients were more often smokers (p<0.001) and alcohol users (p<0.001). They were more often overweight and obese with a body mass index (BMI) (46) greater than 25 (p<0.001). Men also showed a more advanced tumor stage compared to women (p=0.01), therefore leading to more adjuvant treatments: indeed, 45.1% of men had RT (p=0.002).

No statistically significant differences between the two sexes were found upon considering tumor family history, adjuvant chemotherapy or NLR.

In both univariate and multivariate analysis no significant OS differences were highlighted (p=0.61 and p=0.34, respectively). Women presented a median survival of 11y and men of 13.3y.

Low hemoglobin levels were found to be significantly associated with OS only for women (p=0.007), showing more than 2 times greater risk of death (HR =2.66, 95%CI 1.50-4.70). RT treatment and DOI ≤ 10 resulted as independent positive prognostic factors in the multivariate models only for women while vascular invasion and perineural infiltration were significant only for men (**Table 2**).

**Table 2:** Overall Survival (OS) for 576 patients, multivariate analysis.

		Subgroup analysis	
	P value*	Women HR (95%CI), p	Men HR (95%CI), p
In general population:			
M vs W	0.34		
Subgroup:			
Age		**1.03 (1.02-1.04), <0.001**	**1.03 (1.01-1.04), <0.001**
Stage VIII edition			
Stage II vs I		1.03 (0.53-2.00), 0.93	0.87 (0.38-1.98), 0.74
Stage III and IV vs I		0.64 (0.26-1.57), 0.33	**2.42(1.24-4.74), 0.01**
Alcohol			
Ever vs never		1.31 (0.83-2.08), 0.24	1.14 (0.78-1.67), 0.49
Hemoglobin level			
Low hemoglobin level vs not		**2.66 (1.50-4.70), 0.007**	1.31 (0.81-2.12), 0.27
NLR			
NLR>2.37 vs ≤2.37		1.20 (0.78-1.84), 0.41	1.37 (0.96-1.95), 0.08
RT performed			
Yes vs not		**0.55 (0.32-0.97), 0.04**	1.04 (0.70-1.55), 0.84
BMI		0.98 (0.95-1.02), 0.47	0.99 (0.95-1.04), 0.81
DOI			
≤10 vs >10mm		**0.25 (0.10-0.60), 0.001**	–
Vascular invasion			
No vs Yes		–	**0.43 (0.24-0.77), 0.005**
Perineural infiltration			
Yes vs No		–	**1.85 (1.17-2.92), 0.008**

*P value of multivariate Cox model adjusted for stage, age, alcohol, hemoglobin level, vascular invasion, NLR and RT performed.

HR, Hazard Ratio; CI, Confidence Interval; M, men; W, women; NLR, neutrophil lymphocyte ratio; RT, radiotherapy; BMI, body mass index; DOI, depth of invasion.

In DFS univariate analysis no significant differences among sexes were found (p=0.84). Women showed a median DFS of 3.91y (2.52y-7.74y) while men of 4.96y (3.55y-7.41y).

In multivariate analysis upon investigating factors associated with DFS, sex was not significantly associated with relapse (p=0.66), while a low hemoglobin level was confirmed to be an independent prognostic factor for women (p=0.006).

Among women, patients with a low hemoglobin level were found to have more than twofold higher risk of relapse (HR=2.09, 95% CI 1.24-3.53).

RT and DOI ≤ 10 resulted as independent positive prognostic factors for women (p=0.009 and p=0.002, respectively) while perineural infiltration only for men (**Table 3**).

**Table 3:** Disease Free Survival (DFS) for 576 patients, multivariate analysis.

		Subgroup analysis	
	P value*	Women HR (95%CI), p	Men HR (95%CI), p
In general population:			
M vs W	0.66		
Subgroup:			
Age		**1.02 (1.01-1.03), 0.002**	**1.02 (1.01-1.04), <0.001**
Stage VIII edition			
Stage II vs I		0.98 (0.56-1.71), 0.95	0.77 (0.41-1.46), 0.43
Stage III and IV vs I		0.67 (0.33-1.37), 0.28	**1.97 (1.16-3.33), 0.01**
Alcohol			
Ever vs never		1.28 (0.85-1.93), 0.24	1.23 (0.88-1.72), 0.21
Hemoglobin level			
Low hemoglobin level vs not		**2.09 (1.24-3.53), 0.006**	1.08 (0.70-1.66), 0.73
NLR			
NLR>2.37 vs ≤2.37		1.35 (0.93-1.97), 0.11	1.16 (0.85-1.58), 0.36
RT performed			
Yes vs not		**0.51 (0.30-0.84), 0.009**	0.74 (0.52-1.07), 0.11
DOI		**0.34 (0.17-0.68), 0.002**	
≤10 vs >10 mm			–
Perineural infiltration		–	
Yes vs no			**2.16 (1.47-3.16), <0.001**

*P value of multivariate Cox model adjusted for stage, age, alcohol, hemoglobin level, vascular invasion, NLR and RT performed.

HR, Hazard Ratio; CI, Confidence Interval; M, men; W, women; BMI, body mass index; NLR, neutrophil lymphocyte ratio; RT, radiotherapy; DOI, depth of invasion.

Smoking and status of margins variables were also tested, but since they were found not statistically significant in the multivariate analysis they were not included in the final models (results not shown).

Age resulted as independent prognostic factor for both sexes for OS and DFS (**Tables 2** and **3**). Upon categorizing age with the median value, the association with tumor outcomes was confirmed (p<0.0001 for OS and DFS, data not shown).

### Stratified analysis for cancer stage

#### Stage I and II

We identified 224 patients with stage I and II OTSCC, 107 (47.8%) women and 117 (52.2%) men.

**Figure 1:**
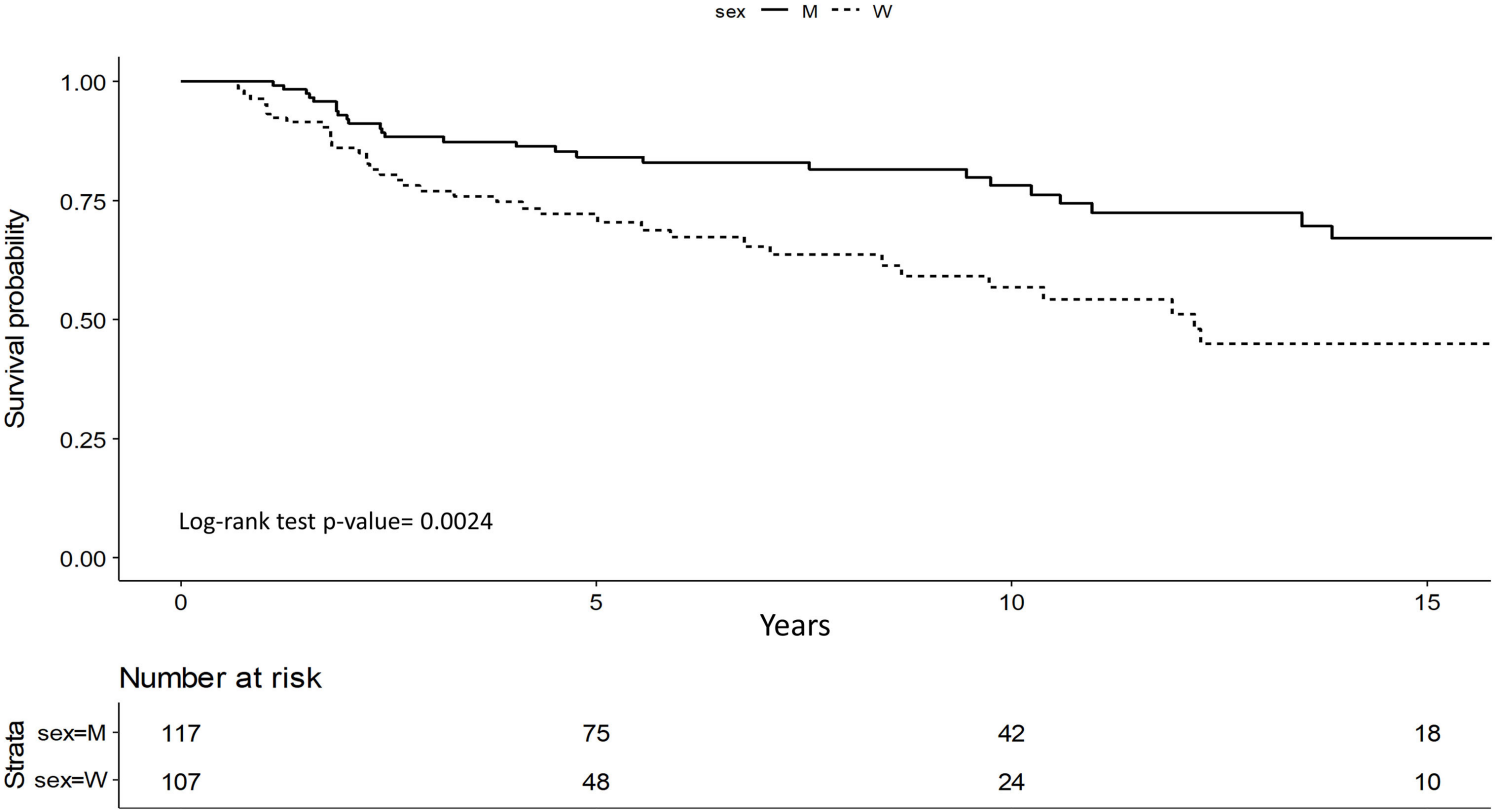
Overall Survival (OS): women vs men in stage I and II.

Survival analysis showed that men have significantly better OS (p=0.002) (**Figure 1**).

This difference was confirmed by multivariate analysis: even upon adjusting for possible confounding factors, S/G appeared to be an independent prognostic factor (p=0.009). In particular, men have a 56% lower risk of death compared to women (HR=0.44; 95%CI 0.24-0.81). Low hemoglobin levels presented a significant impact for women with a worse OS (HR=3.99; 95%CI 1.55-10.2, p=0.004) (**Table 4**).

**Table 4:** Overall Survival (OS) for stage I-II patients, multivariate analysis.

		Subgroup analysis	
	P value*	Women HR (95%CI), p	Men HR (95%CI), p
In general population:			
M vs W	0.009		
Subgroup:			
Age		**1.03 (1.007-1.05), 0.01**	**1.05 (1.01-1.09), 0.005**
Alcohol			
Ever vs never		1.67 (0.80-3.52), 0.17	2.54 (0.83-7.76), 0.11
Hemoglobin level			
Low hemoglobin level vs not		**3.99 (1.55-10.2), 0.004**	3.44 (0.79-14.9), 0.10
NLR			
NLR>2.37 vs ≤2.37		0.99 (0.50-2.00), 0.99	1.96 (0.83-4.62), 0.12
BMI		0.99 (0.91-1.07), 0.78	0.90 (0.78-1.03), 0.13

*P value of multivariate Cox model adjusted for age, alcohol, hemoglobin level, NLR and RT performed.

HR, Hazard Ratio; CI, Confidence Interval; M, men; W, women; NLR, neutrophil lymphocyte ratio; RT, radiotherapy; BMI, body mass index.

**Figure 2:**
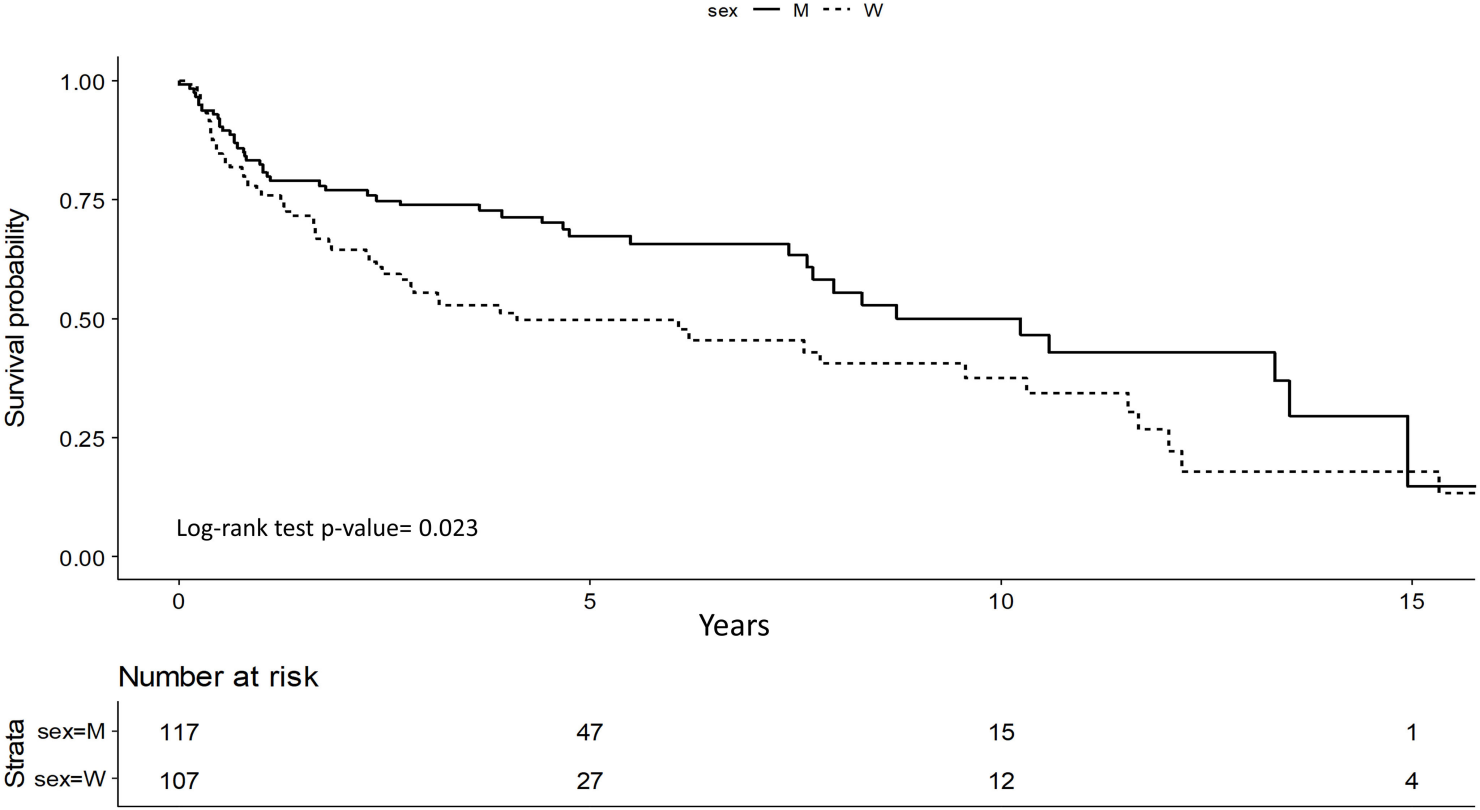
Disease Free Survival (DFS): women vs men in stage I and II.

DFS was better for men with stage I and II (p=0.023) and it was confirmed in multivariate study (p=0.01). Men showed a 45% lower risk of relapse compared to women (HR=0.55; 95%CI 0.34-0.87) (**Figure 2**).

Older age was related to a worse DFS for women (HR=1.03; 95%CI 1.01-1.04, p=0.002), alcohol consumption was a worsening risk factor in both sexes (Women HR=1.89; 95%CI 1.01-3.52, p=0.04; Men HR=2.28; 95%CI 1.07-4.88, p=0.03).

In early cancer stage, low hemoglobin level was significantly associated with relapse in men, but the CI was very wide (HR=6.92; 95%CI 2.30-20.8, p <0.001) (**Table 5**). RT treatment variable was also tested, but as it was found not statistically significant it was excluded from the models (results not shown).

**Table 5:** Disease Free Survival (DFS) for stage I-II patients, multivariate analysis.

		Subgroup analysis	
	P value*	Women HR (95%CI), p	Men HR (95%CI), p
In general population:			
M vs W	0.01		
Subgroup:			
Age		**1.03 (1.01-1.04), 0.002**	1.02 (0.99-1.04), 0.15
Alcohol			
Ever vs never		**1.89 (1.01-3.52), 0.04**	**2.28 (1.07-4.88), 0.03**
Hemoglobin level			
Low hemoglobin level vs not		2.00 (0.87-4.61), 0.10	**6.92 (2.30-20.8), <0.001**
NLR			
NLR>2.37 vs ≤2.37		1.17 (0.65-2.11), 0.60	1.24 (0.65-2.35), 0.51

*P value of multivariate Cox model adjusted for age, alcohol, hemoglobin level, NLR and RT performed. HR, Hazard Ratio; CI, Confidence Interval; M, men; W, women; NLR, neutrophil lymphocyte ratio.

#### Stage III and IV

We analyzed 352 patients with stage III and IV, 123 (34.9%) women and 229 (65.1%) men.

No differences were found between the sexes in term of OS in both univariate and multivariate analysis (p=0.44 and p=0.70, respectively). Similar results were found for DFS (univariate p=0.37; multivariate p=0.23).

For women in advanced stage, NLR > 2.37 (> median) was significantly associated with a worse DFS (HR=1.79; 95%CI 1.07-3.00, p=0.02).

Increasing age was associated with a worse OS in both sexes (OS women HR=1.03; 95%CI 1.01-1.05, p<0.001; men HR=1.03; 95%CI 1.01-1.04; p=0.002). The DOI was a prognostic factor for women but not for men, in particular a DOI <10 mm was associated with better OS and DFS. Perineural infiltration was significant only for men.

For women, low hemoglobin levels were significantly associated with a worse OS (p=0.051) (**Supplementary Table S3**) and RT treatment was significantly associated with a better DFS (p=0.005) (**Supplementary Table S4**).

## Discussion

Our analysis highlighted the S/G differences in terms of prognosis, focusing on early tongue squamous cell carcinoma (OTSCC) stages, on patients’ hemoglobin levels and NLR values.

To our knowledge this study is one of the largest cohort studies investigating the role of S/G on prognosis in a homogenous group of non-metastatic OTSCC patients.

In early tumor stages (I-II), men presented half risk of death and relapse than women, while women had low hemoglobin levels with a statistically worsening of OS and DFS. These differences did not seem to impact advanced tumor states (III-IV), since in these cases the prognosis is already influenced by the late diagnosis of the disease.

The importance of stage as a prognostic value is well known and studied in HNC: patients diagnosed at advanced stages (III-IV) present a 40% 5-year relative survival rate compared to the 85% for patients diagnosed at early stages, according to the 7^th^ TNM edition (14, 47, 48).

Specifically, for oral cancers a new version of the TNM system staging was published in 2018 (8^th^ edition) firstly including well known prognostic factors such as the depth of tumor infiltration (DOI) and the presence of extracapsular tumor spread (ECE) in neck lymph nodes at the diagnosis (41). These data helped clinicians to improve the stratification of prognosis in patients with tongue cancer by better dividing the survivals relative to each stage of the disease. However, the wide difference in oncological outcomes between the early and advanced stages did not change (48–50). Even if the 8^th^ TNM edition better stratifies the prognosis (43, 51), the role of S/G is not considered.

In HNC only approximately 30% of patients are diagnosed at an early stage (48, 52) and, in our cohort, women appear to be the largest group in stages I-II, but they are also the group with the worst prognosis in these stages, both as OS and as DFS.

These findings agree with other studies. Women are generally more concerned about their health and seeking care earlier, therefore potentially enhancing prevention measures (13, 24, 53).

Nowadays, this worse trend in OTCSS in women is not readily explained, since the immune response is generally more robust in women than in men (7).

In stages III-IV our analysis did not underline any S/G differences. The lack of difference in prognosis between men and women in advanced stages of oral cancer has already been noted in other works (24, 54).

Unfortunately, the 5-year relative survival rate decreases from 85% in patients diagnosed in early stages to 40% for those diagnosed with an advanced disease (53, 55). Only approximately 30% of cases have been diagnosed as localized tumors in the last decade (53, 55).

Therefore, it is mandatory to understand these trends in late-stage HNC presentation as well as the associated risk factors, since only few studies examined the incidence trends for tongue tumor late-stage, considering sociodemographic variables, including S/G (20).

In our study women with early stages OTSCC have twice the risk of dying than men, despite the known better prognosis of these patients. Moreover, women with low hemoglobin levels had 2 times worse OS than non-anemic women and a 77% higher risk to present an event in terms of DFS. A meta-analysis showed that anemia is an independent prognostic factor for survival in patients with different cancers, including HNC (23).

Clinically, the reduction of the hemoglobin level is related to tumor presence causing chronic inflammation (21). Oral carcinogenesis is correlated with persistent inflammation through the tumor necrosis factor and the release of pro-inflammatory cytokines.

Earlier studies have shown that more women are often diagnosed with anemia compared to men because of their physiology due to monthly menstrual cycles and because more women adopt vegetarian lifestyles (26, 27, 56). Moreover, in adult men hormone testosterone simulates erythropoietin, boosting hemoglobin concentration (27, 57).

Although not many, there are studies describing the relationship between low hemoglobin levels and OTSCC. The presence of anemia is related to a poor prognosis, increasing the risk of mortality, with a decreased local control of cancer and OS (58, 59). Thus, low hemoglobin levels can be considered a predictor of poorer response to treatment (21, 59). Not only in HNC but also in lung cancer a low pre-operative hemoglobin level was reported to be associated with poorer OS (60, 61).

Currently, many known prognostic factors are related to elements which become evident only after surgery, especially in OTSCC, such as the surgical margins (free or involved by disease), the T-N tract status and ECE lymph nodes (62–64). However, the concept of low hemoglobin levels at time of diagnosis can be used to better stratify patients, especially for women, even before the planned treatments which is currently based only on clinical tumor staging.

Regardless of S/G, our data confirmed that the prognosis of tongue cancers remained significantly associated with age: older age seemed to be a worse prognostic factor for all stages in both sexes (49, 65).

RT treatment and DOI ≤ 10 resulted as independent positive prognostic factors in the multivariate models only for women. In particular, women who performed RT and had DOI (mm) <10 showed a lower risk of death and recurrence. On the contrary, vascular invasion and perineural infiltration were negative prognostic factors only for men in the whole cohort.

These factors are known prognostic factors in oral cancer (66, 67), but, to date, their role has not yet been evaluated in terms of the difference between men and women.

The influence and the role of sex on the activation of the immune system including innate and adaptive mechanisms and on cancer development and suppression is an important area of research. Sex-dependent differences in cancer metabolic pathways and immune response are also being considered and studied to assess whether and how sex could influence cancer prognosis (7, 68).

In our study, only women in advanced stage with an NLR> 2.37 had a 70% more probability of relapse.

Roberts et al. (13) did not report sex-related survival disparities among all HNC. Their sample, albeit large (more than 500 patients), compared and collected data on cancers of the entire head and neck area. However, it is well known that HNC are heterogenous and present many different risks, incidences, and survival rates not only between tumors of the oral cavity, oropharynx, and larynx but also within the same sub-site. In fact, in the oral cavity site, the subsites such as the cheek, the mobile tongue, the lips, have different survival outcomes (24).

In a study on 6830 patients presented oral cavity cancer in stage I-II, sex was reported significant in univariate analysis with a worse outcome for women, but the difference was not reported in multivariate analysis (12).

In another recent study on 2082 oral cavity cancers, no sex-dependent differences in OS and disease specific survival were reported, but the analysis was not divided in stages and oral tongue cancer was a subsite included in the whole cohort (69).

Our study presents some limitations: the data belong to a retrospective and monocentric collection, and it could present problems of sample size and statistical power for the stratified analyzes. Furthermore, as the variables included in the models have been chosen on statistical and clinical/epidemiological criteria, there could be a residual confounding problem. Of note, in a retrospective context it is difficult to assess how specific behaviors, that may differ among genders, affect biological factors related also to sex. Despite this, to our knowledge, it is the first study with complete patients’ information (diagnosis, risk factors, treatment, and follow-up) on a large group of patients (576) affected by oral tongue (a single HNC site) that investigates the difference between men and women in terms of oncological survival outcomes.

Investigating all the genetic, epigenetic, hormonal, and gendered social issues related to both sex and gender is the first step to better studying these tumors understanding the differences in oncological results for OTSCC in the two sexes, if any.

## Conclusion

In the era of personalized medicine, specific studies focusing on the diversity not only among sexes but also genders, will be key for the success of precision treatments, also for tongue cancers. In fact, according to our data among patients with oral tongue carcinoma in early stages, men presented better OS and DFS than women and a low hemoglobin level appeared to be an independent prognostic factor for women. For advanced stages (III-IV) S/G was not found to be a significant factor related to the oncological outcome. Furthermore, increasing age was a worsening element regardless of tumor stage and sex.

Even though hemoglobin levels are not currently taken into account for tailored treatment, in our work we found it as a different variable between women and men. In the future, this value may be considered as an indicator for prognosis sex specific.

## Data availability statement

The raw data supporting the conclusions of this article will be made available by the authors, without undue reservation.

## Ethics statement

The studies involving human participants were reviewed and approved by the Ethics Committee of Istituto Europeo di Oncologia (approval number 225). The patients/participants provided their written informed consent to participate in this study.

## Author contributions

MT drafted the manuscript. OD’E realized statistical analysis and revised the manuscript. RB collected clinical data. OD’E, AG, and CM realized statistical analysis. SC and SG conceptualized the study. FM reviewed histopathological patients’ data. AP, MA, and SC critically reviewed the manuscript for important intellectual content. All authors contributed to the article and approved the submitted version.

## Funding

This work was partially supported by the Italian Ministry of Health with Ricerca Corrente and 5x1000 funds and by Cariplo Foundation Grant no. 2019-3283 to SC.

## Acknowledgments

The Authors thank Donatella Scaglione for her data management support.

## Conflict of interest

The authors declare that the research was conducted in the absence of any commercial or financial relationships that could be construed as a potential conflict of interest.

## Publisher’s note

All claims expressed in this article are solely those of the authors and do not necessarily represent those of their affiliated organizations, or those of the publisher, the editors and the reviewers. Any product that may be evaluated in this article, or claim that may be made by its manufacturer, is not guaranteed or endorsed by the publisher.

## Supplementary material

Supplementary Table 1 Failure events of the whole patients’ cohort.

Supplementary Table 2 Deaths and causes of the whole patients’ cohort.

Supplementary Table 3 Overall Survival (OS) for stage III-IV patients, multivariate analysis.

Supplementary Table 4 Disease Free Survival (DFS) for stage III-IV patients, multivariate analysis.
