# Supplementary material for: The prognostic role of sex and hemoglobin levels in patients with oral tongue squamous cell carcinoma
Source: Front Oncol. 2022 Nov 15;12:1018886. doi: 10.3389/fonc.2022.1018886 (PMC9706199; doi:10.3389/fonc.2022.1018886)
Supplement: Supplementary Table 1 — Failure events of the whole patients’ cohort. [file DataSheet_1.docx]

# Supplementary Tables

**Table S1.** Failure events of the whole patients’ cohort.

| **Failure** | **N (%)** |
| --- | --- |
| Overall | 248 |
| Distant | 38 (15.2) |
| Local | 54 (21.8) |
| Local and distant | 3 (1.2) |
| Local and regional | 14 (5.6) |
| Local, regional and distant | 2 (0.8) |
| Regional | 81 (32.7) |
| Regional and distant | 10 (4.0) |
| Second tumor | 46 (18.7) |

**Table S2.** Deaths and causes of the whole patients’ cohort.

| **Deaths** | **N (%)** |
| --- | --- |
| Overall | 246 |
| Other cause | 30 (12.2) |
| Unknown cause | 32 (13.0) |
| Second tumor | 25 (10.2) |
| Primary tumor | 159 (64.6) |

**Table S3.** Overall Survival (OS) for stage III-IV patients, multivariate analysis.

|  | |  | Subgroup analysis | | |
| --- | --- | --- | --- | --- | --- |
|  | P value* | | | Women  HR (CI 95%), p | Men  HR (CI 95%), p |
| In general population:  M vs W | 0.70** | | |  |  |
| Subgroup:  Age  Alchol  Ever vs never  Hemoglobin level  Low hemoglobin level vs not  NLR  NLR$>$2.37 vs $\leq$2.37 |  | | | 1.03 (1.01-1.05), 0.001  1.17 (0.66-2.09), 0.59  2.20 (0.99-4.86), 0.051  1.44 (0.82-2.54), 0.20 | 1.03 (1.01-1.04), 0.0006  1.06 (0.70-1.61), 0.77  1.18 (0.70-1.98), 0.53  1.22 (0.83-1.79), 0.31 |
| BMI  RT performed  Yes vs not  DOI (mm)  ≤10 vs >10 mm  Vascular invasion  Not vs Yes  Perineural infiltration  Yes vs not |  | | | 0.99 (0.94-1.03), 0.60  0.61 (0.34-1.10), 0.10  0.30 (0.13-0.71), 0.006  -  - | 1.00 (0.96-1.05), 0.89  1.02 (0.68-1.52), 0.93  -  0.42 (0.24-0.76), 0.004   - 1. (1.20-3.01), 0.006 |
|  | | | | | |
| *P value of multivariate Cox model adjusted for age, alcohol, hemoglobin level, vascular invasion, NLR and RT performed. M: men; W: women; NLR: neutrophil lymphocyte ratio, RT: radiotherapy; DOI: depth of invasion.  **HR=1.07 (0.76-1.51)-> M vs W. | | | | | |

**Table S4.** Disease Free Survival (DFS) for stage III-IV patients, multivariate analysis.

|  | |  | Subgroup analysis | | |  |
| --- | --- | --- | --- | --- | --- | --- |
|  | P value* | | | Women  HR (CI 95%), p | Men  HR (CI 95%), p | |
| In general population:  M vs W | 0.23** | | |  |  | |
| Subgroup:  Age  Alchol  Ever vs never  Hemoglobin level  Low hemoglobin level vs not  NLR  NLR$>$2.37 vs $\leq$2.37  RT performed  Yes vs not  DOI (mm)  ≤10 vs >10 mm  Vascular invasion  Not vs yes  Perineural infiltration  Yes vs not |  | | | 1.01 (1.00-1.03), 0.11  1.04 (0.60-1.79), 0.89  1.92 (0.96-3.85), 0.07  1.79 (1.07-3.00), 0.02  0.45 (0.26-0.78), 0.005  0.37 (0.18-0.75), 0.006  0.37 (0.15-0.90), 0.03  - | 1.02 (1.01-1.04), <0.001  1.09 (0.75-1.58), 0.66  0.87 (0.54-1.41), 0.57  1.12 (0.79-1.59), 0.52  0.75 (0.52-1.08), 0.12  -   - 1. 0.33-0.96), 0.03   2. (1.25-2.90), 0.002 | |
| *P value of multivariate Cox model adjusted for age, alcohol, hemoglobin level, vascular invasion, perineural infiltration, NLR and RT performed. M: men; W: women; NLR: neutrophil lymphocyte ratio, RT: radiotherapy; DOI: depth of invasion.  ** HR=1.21 (0.88-1.66)-> M vs F. | | | | | | |
